# Racial and Ethnic Disparities in Excess Deaths after COVID-19 Vaccine Deployment among Persons with Kidney Failure

**DOI:** 10.2215/CJN.0000000000000226

**Published:** 2023-06-14

**Authors:** Daeho Kim, Shailender Swaminathan, Yoojin Lee, Virginia Wang, Rajnish Mehrotra, Amal N. Trivedi

**Affiliations:** 1 Department of Health Services, Policy and Practice, Brown University, Providence, Rhode Island; 2 Sapien Labs Centre for Human Brain and Mind, Krea University, Chennai, India; 3 Department of Population Health Sciences, Duke University School of Medicine, Durham, North Carolina; 4 Department of Medicine, Duke University School of Medicine, Durham, North Carolina; 5 Durham Center of Innovation to Accelerate Discovery and Practice Transformation, Durham Veterans Affairs Health Care System, Durham, North Carolina; 6 Department of Medicine, University of Washington School of Medicine, Seattle, Washington; 7 Providence VA Medical Center, Providence, Rhode Island

**Keywords:** COVID-19, ESKD, health equity, diversity, and inclusion, mortality

## Introduction

Coronavirus disease 2019 (COVID-19) has resulted in stark racial/ethnic disparities in excess deaths among persons with kidney failure.^1^ We previously reported that 72% excess deaths among patients with kidney failure occurred among Black and Hispanic patients from March to July of 2020, and the relative increase in excess deaths observed among Black and Hispanic patients was more than four-fold higher than among White patients. It is unclear how these disparities have changed over the course of the pandemic, particularly after the deployment of COVID-19 vaccines. This analysis assessed disparities in excess mortality for the Medicare population with kidney failure from March 1, 2020, through December 31, 2021.

## Methods

Data on deaths and race/ethnicity (non-Hispanic White, non-Hispanic Black, Hispanic, or other) for the kidney failure population from 2015 through 2021 were obtained from the Research Triangle Institute race variable in the Medicare Beneficiary Summary File.

We estimated expected deaths during the COVID-19 period (March 1, 2020, to December 31, 2021) by fitting a Poisson regression model to the pre–COVID-19 data (January 4, 2015, to February 29, 2020), with adjustments for linear time trend, year fixed effects, and seasonality using Fourier terms. We then calculated excess deaths—*i.e.*, additional number of deaths beyond what was expected on the basis of the fitted historical trends. After previous research characterizing the proportion of excess deaths attributable to the pandemic using expected deaths as the denominator, we also calculated percent excess deaths (a.k.a., P-score).^2^ Separate Poisson models were applied to each racial/ethnic group to compute weekly and cumulative expected deaths, excess deaths, and percent excess deaths before and after the wide deployment of COVID-19 vaccines (before and after January 31, 2021). Brown University's Institutional Review Board approved the analyses.

## Results

**Figure 1:**
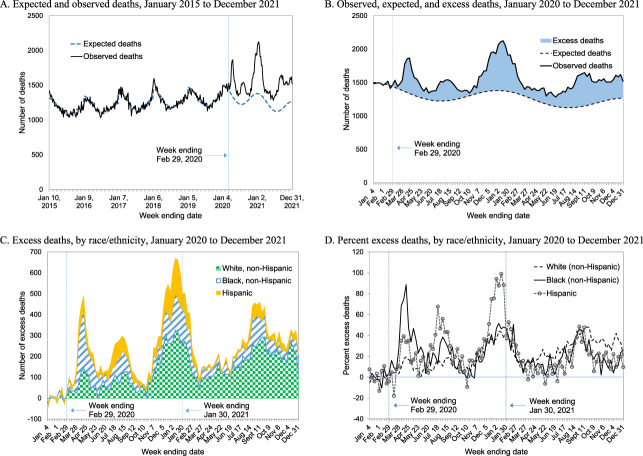
**Excess deaths among patients with kidney failure.** (A) Expected and observed deaths, January 2015 to December 2021. (B) Observed, expected, and excess deaths, January 2020 to December 2021. (C) Excess deaths, by race/ethnicity, January 2020 to December 2021. (D) Percent excess deaths, by race/ethnicity, January 2020 to December 2021. (A–C) The observed deaths (solid line) are weekly number of deaths; the expected deaths (dotted line) are estimated from a Poisson regression; and the area between the observed and expected deaths depicts the number of excess deaths. (D) The percentage of excess deaths calculated by dividing excess deaths by expected deaths (baseline).

There were 686,719 patients with kidney failure in January 2020. Figure 1A shows weekly numbers of expected and observed deaths from January 2015 through December 2021, showing that the Poisson model performs well—*i.e.*, expected deaths predicted from the model match closely with observed deaths before the pandemic (January 2015 to February 2020). Figure 1B shows an increase in excess deaths beginning March 1, 2020, with a peak in January 2021. From March 1, 2020, through January 30, 2021, there were substantial disparities in excess deaths across racial/ethnic groups (Figure 1, C and D). The number of excess deaths was 5582 (95% confidence interval [CI], 4717 to 6450), 4303 (95% CI, 3682 to 4931), and 2679 (95% CI, 2181 to 3177) for non-Hispanic White, non-Hispanic Black, and Hispanic patients, respectively. The percent excess deaths was 31.9% (95% CI, 24.0 to 39.8) for Hispanic patients, 27.5% (95% CI, 22.5 to 32.4) for non-Hispanic Black patients, and 16.4% (95% CI, 13.4 to 19.3) for non-Hispanic White patients. After the wide distribution of COVID-19 vaccines since the end of January 2021, the lowest percent excess deaths was observed among Hispanic patients (17.4% [95% CI, 5.6 to 29.2]), followed by Black patients (19.4% [95% CI, 11.4 to 27.3]) and White patients (27.6% [95% CI, 21.1 to 34.2]).

## Discussion

This study of excess deaths among the kidney failure population showed that during the early stages of the pandemic until January 2021, half of all excess deaths occurred among Black and Hispanic patients, with the highest relative increase in deaths among Hispanic patients. However, these disparities were substantially reduced and indeed reversed after the end of January 2021, when COVID-19 vaccines were widely distributed, with a percent excess deaths of 17.4% among Hispanic patients, 19.4% among Black patients, and the highest (27.6%) among White patients. Possible reasons for the attenuated disparities include a higher take-up rate of vaccines among racial/ethnic minority compared with White patients. A recent study showed higher vaccination rate among Hispanic patients than White patients, with similar rates between Black and White patients in dialysis facilities.^3^

It is noteworthy that the absolute number of excess deaths still remained high (2926 for Black patients and 1377 for Hispanic patients) even after the deployment of vaccines. This finding could result from indirect influences of COVID-19, such as disruption of care and staffing shortage. Our study has several limitations. First, the data cannot distinguish deaths directly due to COVID-19 infection from those due to factors indirectly related to COVID-19. Second, the study population was restricted to Medicare beneficiaries. Third, alternative modeling assumptions may yield different estimates. The findings suggest that racial/ethnic disparities in excess deaths among patients with kidney failure were attenuated after January 2021, when COVID-19 vaccines became widely available and federally supplied vaccines were distributed to dialysis facilities.^4,5^ Despite the reduced disparities, the number of excess deaths among all racial/ethnic groups remained high in the postvaccination period, highlighting the urgent need for additional strategies to address the ongoing adverse effects of the pandemic among persons with kidney failure.
